# Novel and Simple Ultrasonographic Methods for Estimating the Abdominal Visceral Fat Area

**DOI:** 10.1155/2017/8796069

**Published:** 2017-08-22

**Authors:** Takeharu Asano, Naoto Kubota, Norihiro Koizumi, Kazunori Itani, Tsuyoshi Mitake, Kazuhito Yuhashi, Hongen Liao, Mamoru Mitsuishi, Shigemi Takeishi, Toshiaki Takahashi, Shin Ohnishi, Shiro Sasaki, Ichiro Sakuma, Takashi Kadowaki

**Affiliations:** ^1^Department of Gastroenterology, Saitama Medical Center, Jichi Medical University, Saitama, Japan; ^2^Translational Systems Biology and Medicine Initiative (TSBMI), University of Tokyo, Tokyo, Japan; ^3^Department of Diabetes and Metabolic Diseases, Graduate School of Medicine, University of Tokyo, Tokyo, Japan; ^4^Graduate School of Engineering, University of Tokyo, Tokyo, Japan; ^5^Graduate School of Informatics and Engineering, University of Electro-Communications (UEC), Tokyo, Japan; ^6^Hitachi Ltd., Tokyo, Japan; ^7^Department of Gastroenterology, Graduate School of Medicine, University of Tokyo, Tokyo, Japan; ^8^Department of Biomedical Engineering, School of Medicine, Tsinghua University, Beijing, China; ^9^Institute of Rural Medicine, Hiraka General Hospital, Yokote, Akita, Japan

## Abstract

**Objectives:**

To evaluate the abdominal visceral fat area (VFA), we developed novel ultrasonographic (US) methods for estimating.

**Methods:**

100 male volunteers were recruited, and their VFA was calculated by two novel US methods, the triangle method and the ellipse method. The VFA calculated by these methods was compared with the VFA calculated by CT.

**Results:**

Both the VFA calculated by the triangle method (*r* = 0.766, *p* < 0.001) and the ellipse method (*r* = 0.781, *p* < 0.001) showed a high correlation coefficient with the VFA calculated by CT. Also, the VFA calculated by our novel methods were significantly increased in subjects with one or more metabolic risk factors than in those without any risk factors. Furthermore, the correlation coefficients obtained using the two methods were enhanced by the addition of multiple regression analysis (with the triangle method, *r* = 0.8586, *p* < 0.001; with the ellipse method, *r* = 0.8642, *p* < 0.001).

**Conclusions:**

The VFA calculated by the triangle or ellipse method showed a high correlation coefficient with the VFA calculated by CT. These US methods are easy to use, they involve no radiation exposure, and the measurements can be conducted frequently. We hope that our simple methods would be widely adopted for the evaluation of VFA.

## 1. Introduction

Obesity is a major public health problem, the prevalence of which has increased worldwide [1]. Obesity is reported to be associated with type 2 diabetes, cardiovascular disease, nonalcoholic fatty liver disease, and elevated cancer death risk and is a strong predictor of increased morbidity and mortality [2–5]. Furthermore, insulin resistance associated with obesity causes not only type 2 diabetes but also dyslipidemia and hypertension, resulting in the so-called metabolic syndrome [6, 7]. It is, therefore, of pivotal importance to evaluate and treat obesity, in particular, the accumulation of visceral fat [8–10].

Several methods for estimation of the visceral fat area (VFA) have been reported. Until now, computed tomography (CT) is considered as the gold standard method for the measurement of VFA [11–14], although it is expensive and is associated with the risk of radiation exposure at the level of about 10–20 mSv per scan. Waist circumference has been demonstrated to show excellent correlation with the VFA, is measured easily, is noninvasive, and is at low cost [15–19]. Waist circumference represents the VFA and subcutaneous fat area (SFA) as a unit, and it is difficult to distinguish between the two based on the waist circumference alone. Bioelectrical impedance analysis is an easy and noninvasive method that measures the electrical potential difference [20–23]. However, it is difficult to calculate the VFA directly by this method, and it could entail errors associated with water adhesion or change in the total body water amount. None of the methods, including the waist circumference, body mass index (BMI), or bioelectrical impedance analysis, is useful to distinguish between the visceral fat and subcutaneous fat. Magnetic resonance imaging (MRI) allows radiation exposure to be avoided but is expensive, time-consuming, and can be used at only a limited number of institutions [24–27]. Ultrasonography (US) is an easy, inexpensive, and noninvasive method. Some US methods for estimating the VFA have been reported, which measured various body segments or the ratio of subcutaneous and visceral fat [28–33]. Also, due to the problems of ambiguous images, proficiency of procedure, and intrarater and interrater reliability, the preexisting US-based methods are still unsatisfactory and need further improvements.

The purpose of this study was to develop two novel US methods for estimation of the abdominal VFA. Then, multiple regression analysis was performed using several physiological parameters as covariates in order to identify the parameters that would significantly enhance the correlation with the VFA calculated by CT.

## 2. Materials and Methods

For easier and more accurate estimation of the VFA, we devised two novel US methods: the triangle method and the ellipse method. In the first, the triangle method, the VFA is assessed as a summed area of six triangles, and in the second, the ellipse method, the VFA is assessed as part of an ellipse. The VFA calculated by US was compared with the VFA calculated by CT.

### 2.1. The Triangle Method

**Figure 1:**
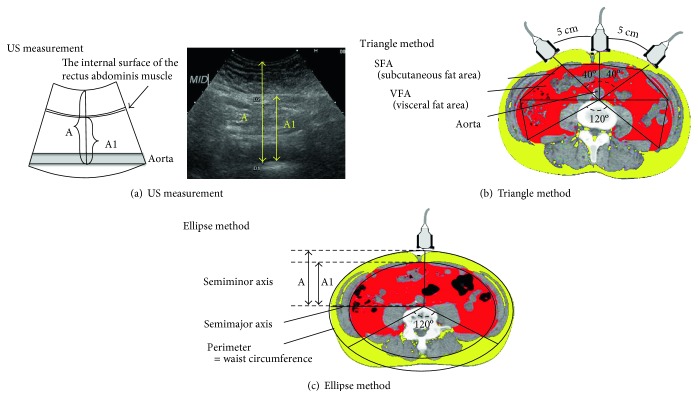
(a) US measurement picture; distance A showed the distance from the skin to the posterior wall of the aorta. Distance A1 showed the distance from the internal surface of the rectus abdominis muscle to the posterior wall of the aorta. The thickness of the subcutaneous fat layer showed the distance from the skin to the rectus abdominis muscle. (b) Triangle method; VFA was calculated as a summed area of the six triangles, which is calculated by the distance to the back wall of the aorta from three positions. (c) Ellipse method; we hypothesized that the ellipsoidal-shaped peritoneal cavity was the reduced scale model of the ellipsoidal-shaped cross section of abdomen. We defined waist circumference as the circumference of the ellipse, and the US-measured distance as the semiminor axis. The VFA was taken by subtracting the back side one-third area occupied by bone and muscles from the peritoneal cavity ellipse.

Since a US probe cannot be placed easily on the surface of the umbilicus, and the aorta is normally located to the left of the center, a point 2 cm to the left of the umbilicus was used as the basal point. Two points, each 5 cm to the left and right of the basal point, were also added, and the measurements were conducted with the US probe placed at these three points. US-determined visceral fat distance was defined as the distance between the internal surface of the rectus abdominis muscle and the posterior wall of the aorta from each diagnostic position (Figure 1(a)). The US-determined VFA was calculated as a summed area of six triangles, which is calculated by the distance to the back wall of the aorta from each position (Figure 1(b)), minus the area of the intestinal tract (10 cm^2^). The area of the intestinal tract was set as 10 cm^2^ based on the average in the CT images of 213 cases, determined in a previous study [34].

We designed a belt-shaped ultrasound probe-compatible device to provide a quick, easy-to-operate, and accurate way to guide the ultrasonographic procedures in the triangle method (Supplementary Information available online at https://doi.org/10.1155/2017/8796069). The belt-shaped device was designed to be fixed in the same positions and angles with respect to the ultrasound probe in different patients. The belt-shaped device has three holes, a center basal point and each 5 cm distant right and left side, for applying the US probe. The angles between the line from the aorta to the basal point and the lines from the aorta to the bilateral holes were each 40° in unbent situation. Made of an elastic material, this belt-shaped device can be bent smoothly to fit the patient's abdomen. We previously reported the parts of these methods in other subjects [35]; we then developed more sophisticated methods and investigated the relation with metabolic risk factors at this time.

### 2.2. The Ellipse Method

Based on the recognition that the peritoneal cavity is ellipsoidal in shape, we hypothesized that the ellipsoidal-shaped peritoneal cavity was the reduced scale model of the ellipsoidal-shaped cross section of the abdomen. We defined waist circumference as the circumference of the cross section of the abdomen and the semiminor axis as the US-measured distance from the skin to the posterior wall of the aorta (Figure 1(c)). The semimajor axis could be estimated from the aforementioned circumference and the semiminor axis. The cross section area of the abdomen was calculated from the semimajor axis and semiminor axis.

The circumference of the peritoneal cavity ellipse was calculated from the waist circumference and the ratio of the measured distances. The distance from the internal surface of the rectus abdominis muscle to the posterior wall of the aorta (distance A1) and the distance from the skin to the posterior wall of the aorta (distance A) were used for the calculation. The area of the peritoneal cavity ellipse was calculated based on the distance from the internal surface of the rectus abdominis muscle to the posterior wall of the aorta as the semiminor axis and the calculated circumference.

The back side one-third area was occupied by bone and muscles; therefore, this area was subtracted from the area of the peritoneal cavity ellipse. Then, the area of the intestinal tract (10 cm^2^) was subtracted, to finally calculate the VFA.

### 2.3. Intrarater and Interrater Reliability

To assess the intrarater and interrater reliability of the US measurements, we carried out a preliminary study on other subjects than the study subjects. This trial was carried out by two highly skilled sonographers in three male volunteers. The measurements were carried out 4 times per day on each man, on two different days. The interclass correlation coefficient (ICC) of the intrarater reliability was calculated by US measurements carried out several times in a subject by an expert US technician (Table 1). The interrater reliability was assessed by 2 expert US technicians. The distance from the skin to the posterior wall of the aorta (distance A shown in Figure 1(a)) and that from the internal surface of the rectus abdominis muscle to the posterior wall of the aorta (distance A1 in Figure 1(a)) were measured as the US-measured distances.

### 2.4. Prospective Study

We recruited 100 volunteer males aged≧20 years at Hiraka General Hospital (Yokote, Akita, Japan) for this study. Subjects who had received treatment for dyslipidemia and/or diabetes mellitus were excluded. Blood samples were drawn in the morning after the subjects had fasted for 12 h, and the serum lipids, fasting blood sugar, and plasma insulin levels were determined. The height and body weight, waist circumference, and blood pressure of the subjects were measured. The waist circumference was measured at the level of the umbilicus with the subject in the standing position in accordance with the Japanese criteria of metabolic syndrome [15].

In this study, four risk factors (high blood pressure, high triglyceride, low high-density lipoprotein (HDL) cholesterol, and hyperglycemia) defined in the criteria of the National Cholesterol Education Program's Adult Treatment Panel III guidelines in 2005 [36], but not the waist circumference, were defined as metabolic risk factors. Subjects currently receiving treatment for dyslipidemia, hypertension, and/or diabetes were also regarded as having the respective risk factors, regardless of the biochemical values.

VFA was calculated by the two ultrasonographic (EUB-8500, Hitachi Ltd., Tokyo, Japan) methods, the triangle method and the ellipse method. The following parameters were measured with a 3.5 MHz convex array probe: (1) the distance from the skin to the posterior wall of the aorta, (2) the thickness of the subcutaneous fat layer, and (3) the distance between the internal surface of the rectus abdominis muscle and the posterior wall of the aorta. Imaging was performed at the end of a normal expiration in the supine position. The US probe was placed against the skin as lightly as possible to prevent compression of the fat layers. The time required for US measurement was within one or two minutes. All the US measurements were carried out in duplicate by the same investigator, an expert US technician.

CT equipment from Toshiba Medical Systems (Tokyo, Japan) was used for the abdominal CT. Imaging was carried out at the end of expiration at the level of the umbilicus in the supine position. The scan interval was set at 7.5 mm. Standard and appropriate measurement methods for waist circumference are different by the country and race. We measured the waist circumference by the level of the umbilicus in accordance with the Japanese criteria of metabolic syndrome [15]. We compared the waist circumference with VFA calculated by CT and then took CT by the level of the umbilicus.

The CT images were analyzed using the Fat Scan ver.4 software (East Japan Institute of Technology Co., Ltd., Hitachi, Ibaraki, Japan) to calculate the abdominal VFA.

The study protocol conformed to the ethical guidelines of the 1975 Helsinki Declaration and was conducted with the approval of the ethics committee of each of the University of Tokyo, Hitachi Ltd., and Hiraka General Hospital. Written informed consent was obtained from each subject for participation in the study.

### 2.5. Statistics

Pearson's correlation coefficients were calculated to assess the association among the clinical parameters and the VFA calculated by CT. The statistical significance of differences in the continuous data between groups was examined by ANOVA. Statistical significance was set at *p* < 0.05.

### 2.6. Multiple Regression Analysis by the Cross Validation Method

To enhance the accuracy of estimation of the VFA, multiple regression analysis was conducted. The parameters used for the analysis along with the new method were the height, weight, BMI, age, and waist circumference. It was needed that the data, which is applied regression method, should be normally distributed. So, as the first step, we confirmed whether the objective variable that is VFA is normally distributed by using the Kolmogorov-Smirnov test. Since the *p* value as its result was 0.7633, the alternative hypothesis, which is “Variable does not follow a normal distribution.”, was rejected. Then, we performed multiple regression analysis using all of the explanatory variables to obtain a regression formula to determine the correlation coefficient. If a better correlation coefficient was obtained, we optimized the formula by eliminating some variables using the stepwise method based on Akaike's Information Criterion (AIC). With respect to the value of the correlation coefficient, we used a 10-fold cross validation to get more accurate results. We conducted the statistical analyses using R 2.13.2 (the R foundation for Statistical Computing) and Psych (Revelle, W. (2012): Procedure for Personality and Psychological Research, Northwestern University, Evanston, Illinois, USA). To make the Kolmogorov-Smirnov test, we used the “ks.test” function. And the function “lm” which is a function to calculate a linear regression coefficient and also linear multiple regression coefficients was used. These functions are included in the default package of R 2.13.2. Psych which is a package to calculate ICC which is aforementioned.

## 3. Results

### 3.1. Validity Testing of the Triangle Method and Ellipse Method

The characteristics of the 100 male study participants are shown in Table 2. Blood tests, physical examination, CT, and US were performed for all the subjects. The average age was 39.6 ± 11.0 years. The average waist circumference was 84.4 ± 9.2 cm, the BMI was 23.6 ± 3.3, and the VFA calculated from the CT images was 79.4 ± 39.1 cm^2^. Only 3 participants were receiving medication for hypertension, while none of the patients were receiving medication for dyslipidemia or diabetes. Most of the participants of this study group were nonobese and had normal glucose tolerance. We assessed the patients for 4 risk factors of the metabolic syndrome, namely, high blood pressure, high triglyceride, low HDL cholesterol, and hyperglycemia. Of the 100 males, 41 had no risk factor, 31 had one risk factor, and 28 men had ≧2 risk factors.

### 3.2. VFA Calculated by the Triangle Method as well as That Calculated by the Ellipse Method Showed a High Correlation Coefficient with the VFA Calculated by CT

**Figure 2:**
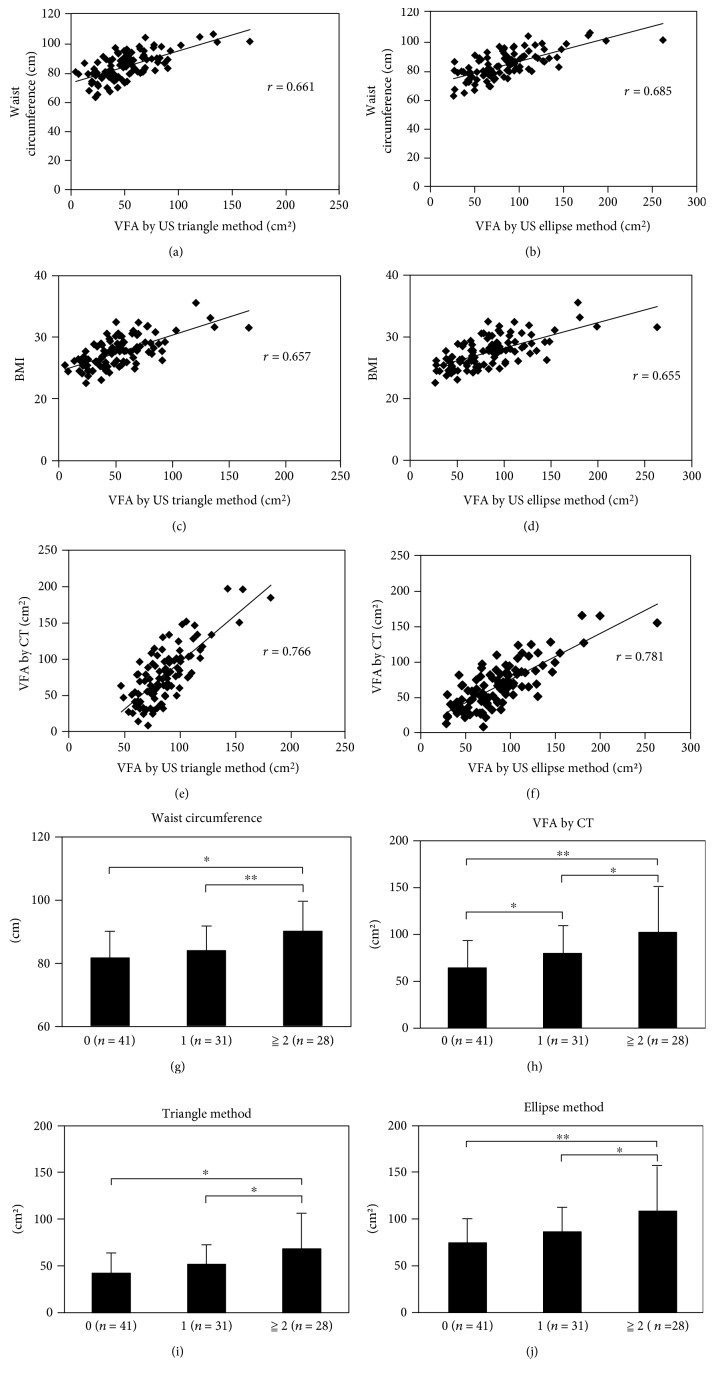
The correlations between the VFA calculated by US method and each of the parameters are shown. (a) Triangle method and waist circumference (*r* = 0.661). (b) Ellipse method and waist circumference (*r* = 0.685). (c) Triangle method and BMI (*r* = 0.657). (d) Ellipse method and BMI (*r* = 0.655). (e) Triangle method and VFA by CT (*r* = 0.766). (f) Ellipse method and VFA by CT (*r* = 0.781). The correlations between the risk factors for metabolic syndrome and each parameter are shown. (g) Waist circumference. (h) VFA by CT. (i) Triangle method. (j) Ellipse method. ^∗^*p* < 0.05, ^∗∗^*p* < 0.01.

The correlations between each parameter of the subjects and the VFA calculated by US are shown in Figures 2(a), 2(b), 2(c), 2(d), 2(e), and 2(f). The VFA calculated by the triangle method was significantly correlated with the waist circumference (*r* = 0.661, *p* < 0.001, Figure 2(a)), BMI (*r* = 0.657, *p* < 0.001, Figure 2(c)), and VFA calculated by CT (*r* = 0.766, *p* < 0.001, Figure 2(e)). Also, the VFA calculated by the ellipse method was significantly correlated with the waist circumference (*r* = 0.685, *p* < 0.001, Figure 2(b)), BMI (*r* = 0.655, *p* < 0.001, Figure 2(d)), and VFA calculated by CT (*r* = 0.781, *p* < 0.001, Figure 2(f)). The values that are regression coefficient (*r*) and significant probability (*p* value) were calculated by “lm” function in R. Among the correlations, the VFA calculated by the ellipse method showed the strongest positive correlation with the VFA calculated by CT.

We next assessed the correlation between each parameter and the 4 risk factors for metabolic syndrome, namely, hypertension, hyperglycemia, and dyslipidemia. The waist circumference was significantly increased in the males with ≧2 risk factors as compared to that in the males without any risk factors (Figure 2(g)). The average waist circumference of the males with ≧2 risk factors was about 90 ± 9.5 cm. The VFA calculated by CT (Fat Scan) was significantly higher in the males with ≧2 risk factors than in the males with no risk factors (Figure 2(h)). The average VFA calculated by CT of the males with ≧2 risk factors was about 102 ± 49 cm^2^. The VFA calculated by each of the triangle method and the ellipse method was similarly significantly increased in the males with ≧2 risk factors than in the males with no risk factors (Figures 2(i) and 2(j)).

### 3.3. Multiple Regression Analysis Was Effective to Enhance the Correlation Coefficients Determined above

**Figure 3:**
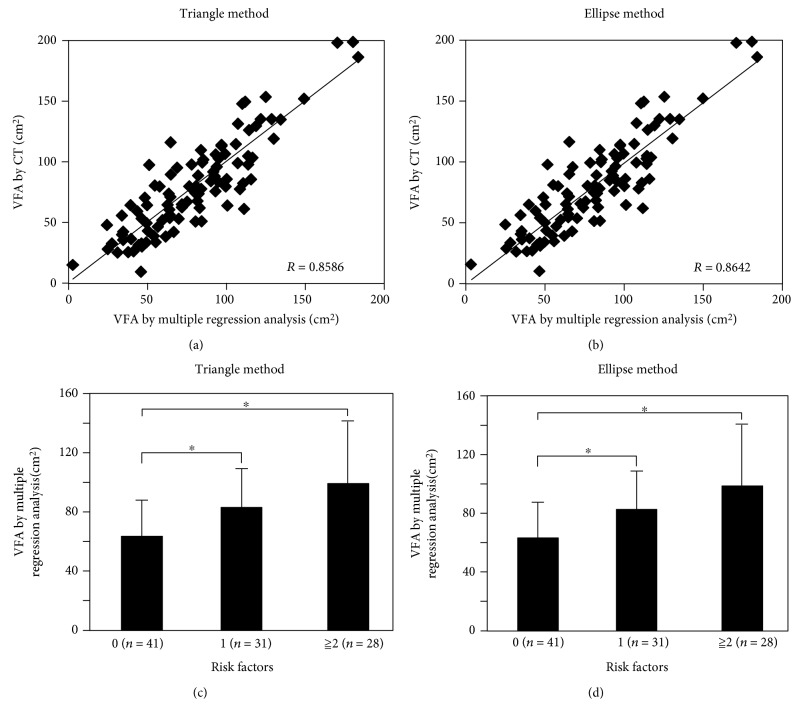
(a) Multiple regression analysis with the triangle method (*r* = 0.8586) and (b) with the ellipse method (*r* = 0.8642). (c) The correlations between the 4 risk factors of metabolic syndrome and the VFA calculated by the triangle method and (d) ellipse method with multiple regression analysis. ^∗^*p* < 0.05.

We used multiple regression analysis to assess the associations between the VFA calculated by CT and several physiological parameters. The results of multiple regression analysis carried out with the triangle method using the height, weight, age, waist circumference, and BMI are shown in Figure 3(a) and Table 3(a). The correlation coefficient obtained by this multiple regression analysis carried out using the aforementioned covariates was 0.8528. The results of the multiple regression analysis carried out with the ellipse method using the height, weight, age, waist circumference, and BMI are shown in Figure 3(b) and Table 3(b). The correlation coefficient obtained by this multiple regression analysis carried out using the aforementioned covariates was 0.8581.

We carried out further multiple regression analysis to investigate which combination would show the best correlation with the VFA calculated by CT (Tables 3(a) and 3(b)). The highest correlation coefficient obtained using multiple regression analysis in the triangle method was 0.8586, using weight, age, and waist circumference as covariates. On the other hand, the highest correlation coefficient obtained using multiple regression analysis in the ellipse method was 0.8642, using age and BMI as covariates. The best regression formula was obtained with the application of the stepwise method in the ellipse method, with age and BMI used as covariates. Since the q-q plot of residual error is located on a straight line, normality was confirmed and *p* value of *t*-test was less than 2.2e−16. Therefore, the regression formula derived seemed valid. In both methods, age was considered as an important factor increasing the correlation coefficient. In fact, multiple regression analysis using age as a covariate is shown in the upper columns in Tables 3(a) and 3(b).

To confirm the reliability of VFA estimation by using triangle method and ellipse method, we applied paired *t*-test for both results. In this test, the alternative hypothesis was “True difference in means not equal to 0.” The *p* values were 0.9646 and 0.9185 for triangle method and ellipse method, respectively. Therefore, the alternative hypothesis should be rejected. And their 95 percent confidential intervals were −4.061 to +3.883 and −4.100 to +3.697, and the mean of the differences were −0.0891 and −0.2015 for each. This analysis showed that both of US estimation methods showed good agreement with VFA measured by CT, in addition to their good correlation values which were aforementioned.

We assessed the correlation between the results of the multiple regression analysis and the 4 risk factors for metabolic syndrome. The VFA calculated using multiple regression analysis with the triangle method was significantly higher in the men with any risk factors than in those with no risk factors (Figure 3(c)). Similarly, the VFA calculated using multiple regression analysis with the ellipse method was increased in the men with any risk factors (Figure 3(d)).

## 4. Discussion

In this study, we describe two novel US methods for estimation of the abdominal VFA. The VFA calculated by the triangle method as well as that determined by the ellipse method showed a high correlation coefficient with the VFA calculated by CT. The VFA calculated by each of these methods was significantly increased in the men with one or more metabolic risk factors than in those with no risk factors. In addition, VFA calculated by these two methods with multiple regression analysis carried out using several parameters as covariates showed a higher correlation coefficient with the VFA calculated by CT.

The triangle method and the ellipse were revealed to be easy and accurate practical evaluation methods for the assessment of VFA. Although both the VFA values calculated by the triangle method and the ellipse method showed high correlation coefficients with the VFA calculated by CT, each of these methods has its own advantages and disadvantages. The advantage of the triangle method is that the calculation needs only US data without measurement of other parameters such as the waist circumference, although 3 points of measurement are needed, with a high level of skill in the US technique. Furthermore, the operation is slightly complicated owing to the use of the belt-shaped device, and time-consuming. Meanwhile, the ellipse method needs measurement of the waist circumference and the operation time is shorter because only one point of measurement by US is needed. Especially, detection of the internal surface of the rectus abdominis muscle and the posterior wall of aorta from 2 cm to the left side of the umbilicus (basal point) is comparatively easy by US, even in obese subjects. In fact, the intrarater and interrater reliability of US measurement at the basal point were very high, and the intrarater reliability was higher for measurement at the basal point than that at a distance of 5 cm from the basal point. (Table 1; data not shown).

In the multiple regression analysis, the age was an important factor for both the triangle method and the ellipse method to enhance the correlation coefficient with the VFA calculated by CT. Muscle mass and basal metabolic rate are known to decrease with aging, and increase of the body fat percentage without body weight change is known to be common in the elderly. Actually, some cross-sectional studies have reported increased visceral fat in the elderly as compared with that in young people for the same BMI [37–40]. Especially, increased distribution of fat to the trunk has been reported in elderly men and women, with at least 50–60% of whole body fat being mainly distributed in the abdomen. Probably, addition of age as a covariate in the multiple regression analysis might compensate for the amount of VFA that cannot be detected in US images and increase the correlation coefficient. In fact, the bioelectrical impedance analysis also incorporates multiple regression analysis, with age added as a covariate.

In regard to other VFA calculation methods, the VFA calculated by the bioelectrical impedance analysis showed a correlation coefficient *r* of 0.88 with the VFA calculated by CT [22]. In the previously reported US VFA calculation methods, the result of the preperitoneal fat thickness method showed an *r* value of 0.746 with the ratio of the visceral fat to subcutaneous fat area determined by CT [30], and that of the method involving measurement of the distance of three abdominal segments showed a value of *r* = 0.860 with the VFA calculated by CT [31]. Multiple regression analysis revealed a good correlation between the results of our triangle method (*r* = 0.8586) and ellipse method (*r* = 0.86420). Especially, despite the easiest procedure to measure by US, the ellipse method showed high correlation coefficient with VFA calculated by CT. These US methods are expected to be useful for evaluation of the time-course of changes of the VFA during diet therapy, physical exercise, and medical intervention in longitudinal studies. By these US methods, patients on dialysis or with heart failure can also be evaluated independent of the body moisture balance, dehydration, and body weight. Patients taking diuretic drugs or sodium-glucose co-transporter 2 (SGLT2) inhibitors for diabetes can also be evaluated under disordered body water balance. These methods could easily be used by the primary care physician or the medical check-up, instead of CT exposure.

This study had the following limitations. First, the study participants were relatively young male volunteers, and therefore, whether the methods are suitable for other age groups remains unknown. Second, the sample size of this study was small, because it was a pilot study. Further large studies are required to assess the suitability of the triangle and ellipse methods for women, elderly people, and patients with metabolic syndrome. Third, whether the intrarater and interrater reliability would still be maintained with a higher number of examiners is unknown.

In conclusion, we devised fast and accurate ultrasonographic methods for the measurement of VFA. The VFA measured by the triangle method as well as that measured by the ellipse method showed a high correlation coefficient with the VFA calculated by CT. These US methods are easy to use, noninvasive, and do not involve radiation exposure, and the measurements can be carried out frequently. We hope that our simple method would be widely adopted for the evaluation of VFA.

## Supplementary Material

Supplementary Figure: We designed a belt-shaped ultrasound probe-compatible device for the triangle method. The belt-shaped device has three holes, a center basal point and each 5 cm distant right and left side, for applying US probe. The angles between the line from the aorta to the basal point and the lines from the aorta to the bilateral holes were each 40° in unbent situation.

## Figures and Tables

**Table 1:** Intrarater reliability and interrater reliability were assessed by measuring the distances A1 and A shown in Figure 1(a) by US.

Intrarater reliability			Interrater reliability	
The distance A1	Investigator A	Investigator B	The distance A1	Correlation coefficient
ICC (1,1)	0.9817	0.9970	ICC (2,1)	0.9988
ICC (1,3)	0.9954	0.9992	ICC (2,3)	0.9987
The distance A	Investigator A	Investigator B	The distance A	Correlation coefficient
ICC (1,1)	0.9800	0.9971	ICC (2,1)	0.9988
ICC (1,3)	0.9949	0.9993	ICC (2,3)	0.9987

ICC: interclass correlation coefficient.

**Table 2:** Characteristics of the study participants (*n* = 100).

	Average	SD	Minimum	Maximum
Age (yr.)	39.6	11.0	22.0	63.0
Waist circumference (cm)	84.4	9.2	63.0	106.4
Height (cm)	172.1	5.3	158.1	181.7
Weight (kg)	70.0	11.0	47.9	98.0
BMI	23.6	3.3	16.9	34.2
VFA (cm^2^)	79.5	39.0	9.8	198.7
SFA (cm^2^)	141.0	66.8	17.0	337.0
Total adipose tissue (cm^2^)	220.4	94.3	32.3	489.3
SBP (mmHg)	129.5	14.3	104.0	174.0
DBP (mmHg)	80.1	12.0	58.0	119.0
FBS (mg/dL)	90.1	11.6	65.0	135.0
Insulin (*μ*U/mL)	6.5	4.1	1.0	23.0
HOMA-R	1.5	1.0	0.2	5.9
HbA1c (%)	5.7	0.3	5.1	6.9
TC (mg/dL)	200.7	32.1	135.0	287.0
HDL-C (mg/dL)	61.7	18.3	30.5	142.2
LDL-C (mg/dL)	121.6	29.3	69.0	207.0
TG (mg/dL)	156.8	140.6	15.0	744.0
UA (mg/dL)	6.2	1.4	2.6	10.3

BMI: body mass index; SBP: systolic blood pressure; DBP: diastolic blood pressure; VFA: visceral fat area; SFA: subcutaneous fat area; FBS: fasting blood sugar; HOMA-R: homoeostatic model assessment ratio; JDS: Japan Diabetes Society; TC: total cholesterol; HDL: high-density lipoprotein; LDL: low-density lipoprotein; TG: triglyceride; UA: uric acid.

**Table:** (a) Containing triangle method.

Triangle method	Height	Weight	Age	Waist circumference	BMl	Correlation coefficient
○	×	○	○	○	×	0.8586435
○	×	○	○	×	×	0.8582122
○	×	×	○	×	○	0.8565199
○	○	×	○	×	○	0.8559084
○	×	○	○	×	○	0.8555399
○	○	○	○	×	×	0.8551473
○	×	×	○	○	○	0.8547307
○	○	×	○	○	○	0.8544964
○	○	○	○	×	○	0.8544280
○	×	×	○	○	×	0.8543381
○	×	○	○	○	○	0.8540807
○	○	○	○	○	×	0.8540032
○	○	○	○	○	○	0.8528104
○	○	×	○	○	×	0.8500605
○	○	×	×	○	×	0.8073538
○	×	×	×	○	×	0.8048660
○	×	×	○	×	×	0.8029817
○	○	×	×	○	○	0.8026849
○	×	○	×	○	○	0.8026364
○	○	○	×	○	×	0.8025610
○	×	○	×	○	×	0.8019843
○	×	×	×	×	○	0.8018995
○	×	×	×	○	○	0.8017493
○	○	○	×	○	○	0.8006840
○	○	×	○	×	×	0.7993090
○	×	○	×	×	○	0.7984585
○	○	×	×	×	○	0.7982493
○	○	○	×	×	×	0.7974290
○	○	○	×	×	○	0.7969801
○	×	○	×	×	×	0.7878478
○	×	×	×	×	×	0.7586674
○	○	×	×	×	×	0.7512694

**Table:** (b) Containing ellipse method.

Ellipse method	Height	Weight	Age	Waist circumference	SMI	Correlation coefficient
○	**×**	**×**	○	**×**	○	0.8642723
○	**×**	○	○	**×**	**×**	0.8641731
○	○	**×**	○	**×**	○	0.8633550
○	**×**	○	○	**×**	○	0.8630552
○	○	○	○	**×**	**×**	0.8630401
○	**×**	○	○	○	**×**	0.8619440
○	○	○	○	**×**	○	0.8617836
○	**×**	**×**	○	○	○	0.8600242
○	○	**×**	○	○	○	0.8598155
○	○	○	○	○	**×**	0.8595745
○	**×**	○	○	○	○	0.8594486
○	○	○	○	○	○	0.8581494
○	**×**	**×**	○	○	**×**	0.8564621
○	○	**×**	○	○	**×**	0.8529285
○	**×**	**×**	**×**	**×**	○	0.8162489
○	○	**×**	**×**	○	**×**	0.8158341
○	○	**×**	**×**	**×**	○	0.8134919
○	**×**	○	**×**	**×**	○	0.8134300
○	○	○	**×**	**×**	**×**	0.8130791
○	**×**	**×**	**×**	○	**×**	0.8122610
○	○	○	**×**	**×**	○	0.8122025
○	**×**	**×**	**×**	○	○	0.8118373
○	**×**	**×**	○	**×**	**×**	0.8113445
○	○	○	**×**	○	**×**	0.8109480
○	○	**×**	**×**	○	○	0.8108606
○	**×**	○	**×**	○	○	0.8106689
○	○	○	**×**	○	○	0.8093496
○	○	**×**	○	**×**	**×**	0.8076415
○	**×**	○	**×**	○	**×**	0.8066263
○	**×**	○	**×**	**×**	**×**	0.8010528
○	**×**	**×**	**×**	**×**	**×**	0.7731967
○	○	**×**	**×**	**×**	**×**	0.7677818

○: model containing the parameter; ×: model not containing the parameter.
