# Supplementary figures and images for: Novel and Simple Ultrasonographic Methods for Estimating the Abdominal Visceral Fat Area
Source: Int J Endocrinol. 2017 Aug 22;2017:8796069. doi: 10.1155/2017/8796069 (PMC5585558; doi:10.1155/2017/8796069)

## Slide 1
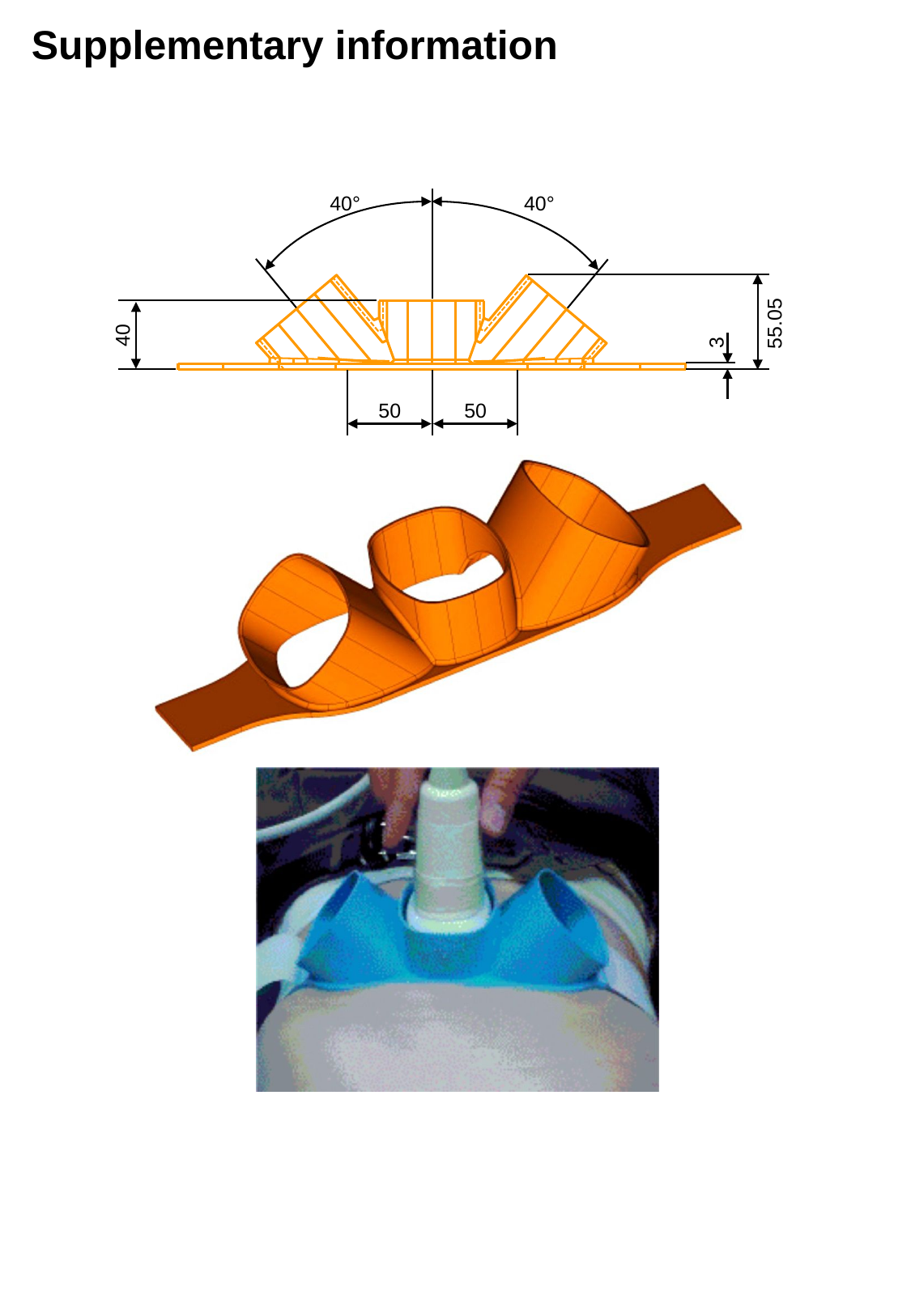

Supplementary information
40°
40°
55.05
40
3
50
50

Supplement: Supplementary file 1 — Supplementary Figure: We designed a belt-shaped ultrasound probe-compatible device for the triangle method. The belt-shaped device has three holes, a center basal point and each 5 cm distant right and left side, for applying US probe. The angles between the line from the aorta to the basal point and the lines from the aorta to the bilateral holes were each 40° in unbent situation. [file 8796069.f1.pptx]
